# One-pot hydrothermal synthesis of Nitrogen-doped graphene as high-performance anode materials for lithium ion batteries

**DOI:** 10.1038/srep26146

**Published:** 2016-05-17

**Authors:** Zheng Xing, Zhicheng Ju, Yulong Zhao, Jialu Wan, Yabo Zhu, Yinghuai Qiang, Yitai Qian

**Affiliations:** 1 School of Materials Science and Engineering, China University of Mining and Technology, Xuzhou, Jiangsu, 221116, P. R. China; 2 Hefei National Laboratory for Physical Science at Microscale, Department of Chemistry, University of Science and Technology of China, Hefei, Anhui, 230026, P.R. China

## Abstract

Nitrogen-doped (N-doped) graphene has been prepared by a simple one-step hydrothermal approach using hexamethylenetetramine (HMTA) as single carbon and nitrogen source. In this hydrothermal process, HMTA pyrolyzes at high temperature and the N-doped graphene subsequently self-assembles on the surface of MgO particles (formed by the Mg powder reacting with H_2_O) during which graphene synthesis and nitrogen doping are simultaneously achieved. The as-synthesized graphene with incorporation of nitrogen groups possesses unique structure including thin layer thickness, high surface area, mesopores and vacancies. These structural features and their synergistic effects could not only improve ions and electrons transportation with nanometer-scale diffusion distances but also promote the penetration of electrolyte. The N-doped graphene exhibits high reversible capacity, superior rate capability as well as long-term cycling stability, which demonstrate that the N-doped graphene with great potential to be an efficient electrode material. The experimental results provide a new hydrothermal route to synthesize N-doped graphene with potential application for advanced energy storage, as well as useful information to design new graphene materials.

In the past decade, graphene has attracted wide attentions due to its fascinating characters and broad applications^1^,^2^ such as in catalysis, water splitting, energy storage and solar cells. These applications of graphene are closely associated with their intrinsic two-dimensional (2-D) crystal structure formed by sp2 hybridized carbon^1^. In the past few years, many researchers have demonstrated that the physical and chemical properties of graphene could be tailored and improved by heteroatom doping on graphene sheets at edges, vacancies, pores and strained regions^3^,^4^. The doping of heteroatoms into the pristine graphene sheets would lead to structural and electronic disorders, which could further result in changes of graphene properties, including Fermi level, bandgap, localized electronic state, thermal stability, electrical conductivity, and magnetic property^5^. New or improved properties could be obtained by altering the type and content of various dopant atoms^6^,^7^.

Nitrogen (1s^2^2s^2^2p^3^)^3^ is the next element to carbon (1s^2^2s^2^2p^2^) in the periodic table with only one less valence electron, which possesses higher electronegativity of N (χ = 3.04) than that of C (χ = 2.55). The doping of N would create polarization in the sp2 carbon network, thereby further influence the chemical and physical properties of graphene. Previous reports demonstrated that N doping is efficient in tuning the characters of graphene materials: the bandgap opening and charge-carrier concentration could be induced by only 0.4 at% doping of graphitic N^8^; pyrrolic N-doping at the edge sites of graphene nanoribbons (GNR) would create strong magnetic moments^9^; the work function of graphene could be reduced by graphitic and pyridinic N-doping^10^,^11^. In this case, nitrogen doping is a useful route for decorating graphene because N-doping easily manipulates local electronic for the potential application including electronic devices^8^, biosensors^12^ and catalysts^13^. Moreover, the nitrogen doped local electronic structures allow for enhanced binding with ions in the solution which is beneficial for lithium ion batteries^14^. Nitrogen-doping would improve the pore affinity to aqueous electrolyte and reduce the electric resistance which would further promote the electrochemical performance of graphene electrode^15^,^16^. For example, nitrogen-doped graphene nanosheets prepared by heat treatment of graphite oxide under an ammonia atmosphere at 800 °C exhibited a high reversible capacity 900 mAh/g at current of 42 mA/g^17^; nitrogen-doped holey graphene hollow microspheres synthesized by a template sacrificing method also exhibited high reversible capacities and rate performances^18^.

To date, N-doped graphene materials can be obtained by various methods including plasma method^14^, arc-discharge approach^19^, chemical vapor deposition (CVD)^20^ and thermal anealing method^21^,^22^. Among these methods, nitrogen-containing functional groups could be doped by reacting with nitrogen-containing reagents (urea, NH_3_ and nitric acid)^23^,^24^ or through carbonizing/activating of nitrogen-rich carbon precursors, such as pentachloropyridine^25^, polypyrrole^26^ and pyrimidine^27^. These methods either require high quality equipment or involve several steps during synthesis process, which are obstacles for large scale fabrication. One step hydrothermal carbonization is a traditional method which is an important technique for the preperation of various carbonaceous materials and hybrids. Recent studies have shown that hydrothermal method is an efficient method for large-scale preparation of graphene: graphene oxide(GO) prepared by oxidation of graphite powder according to the modified Hummers’ method was further reduced by a nitrogen containing reductant and finally transferred into N-doped graphene^28^,^29^.

In this paper, we developed a novel one-step hydrothermal method using HMTA as single carbon and nitrogen source. Briefly, HMTA pyrolyzes in high temperature and the N-doped graphene subsequently self-assembles on the surface of MgO particles (formed by the Mg powder reacting with H_2_O). During this *in-situ* approach, graphene synthesis and nitrogen doping are simultaneously achieved. The as-synthesized graphene with nitrogen-containing functional groups possesses an average thickness of 4–10 nm with interconnected mesoporous system. The high surface area, mesopores and vacancies of the N-doped graphene are particularly beneficial for the reversibly storage of lithium ions. These structural features and their synergistic effects would promote ions and electrons transportation with nanometer-scale diffusion distances as well as be favorable to the penetration of electrolyte. Extensive research carried out on lithium ion batteries demonstrate that the N-doped graphene electrode exhibits high reversible capacity, superior rate capability as well as long-term cycling stability (above 600 mAh/g after 50 cycles at current of 100 mA/g, above 500 mAh/g after 150 cycles at current of 200 mA/g), which suggest its great potential to be an efficient electrode material candidate.

## Results

**Figure 1:**
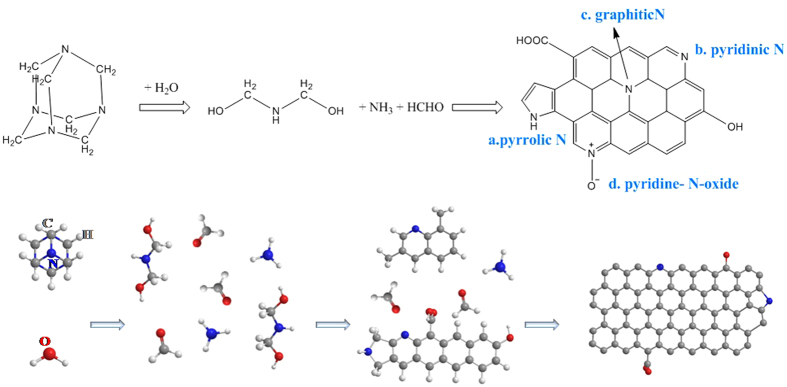
Scheme of a proposed mechanism for the hydrothermal synthesis process from the HMTA molecules to N-doped graphene layer.

Figure 1 illustrates a proposed conversion process from HMTA precursor into N-doped graphene via a hydrothermal synthetic route during which the HMTA is involved in the hydrothermal degradation and recombination and the N-doped graphene is obtained. To give a clear picture, the schematic only focused on the possible changes of the HMTA with H_2_O. Nitrogen atoms appearing at four corners of the cage-like structure are covalently linked with carbon atoms with *sp3* hybridization state in the HMTA molecule precursor which would provide high value nitrogen content. When the hydrothermal treatment is proceeded, the HMTA molecule starts to decompose and hydrolyze into the formation of NH(CH_2_OH)_2_ and release small molecules (CH_2_O, NH_3_)^30^. When the temperature further elevated up, the high temperature induce the structure rearrangement such as of the carbon atoms^31^. The cyclization and conjugation^32^ of the carbon atom lead to the generation and development of two-dimension hexagonal *sp2* carbon clusters^33^, then the freshly formed free *sp2* clusters may assemble into polyaromatic systems and finally into graphene. A variety of nitrogen atoms would residue from the precursors after the whole hydrolyzation and graphitization process; the attachment of the *sp2* carbons to the remaining nitrogen atoms preserves the *sp2* hybridization state in HMTA. Figure 1 illustrates several possible configurations of the nitrogen atoms in graphene: a. pyrrolic N, b. pyridinic N, c. direct substitution (graphitic N) and d. pyridine-N-oxide. Each of these configurations affects the electronic and transport properties of the functionalized material rather differently.

**Figure 2:**
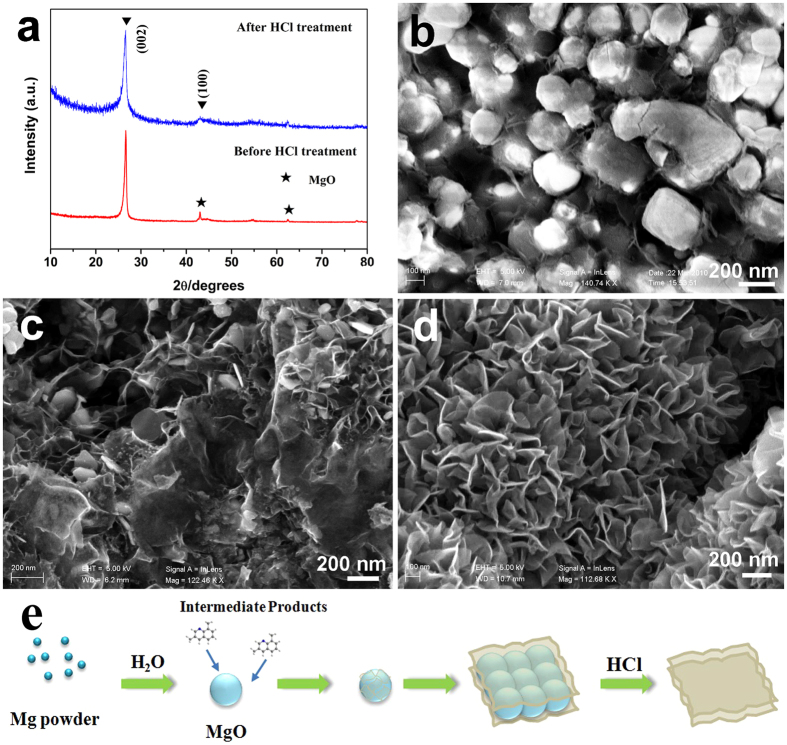
(**a**) XRD patterns of the sample before and after 10 h HCl treatment for graphene; (**b**) SEM images of the raw sample before HCl treatment; (**c**) SEM image of the raw sample after 3 h HCl treatment; (**d**) SEM image of the sample after 10 h HCl treatment; (**e**) Illustration of the formation of graphene nanosheet architectures.

Structural characterization of the as-prepared sample was carried out by the powder X-ray diffraction (XRD). Figure 2a shows the XRD patterns of the product before and after HCl treatment. The XRD pattern of the sample before HCl treatment displays two groups of peaks: the sharp peaks near 2θ = 26° and 43° could be assigned to the (002) and (100) reflections of the 2H phase of graphite (JCPDS No. 41-1487, indicating of graphene layer with d-spacing of 0.34 nm); the peaks around 43° and 62° could be indexed as (111) and (200) crystal face of cubic MgO (JCPDS No. 45-0946). So, the graphene layer and MgO were coexistent in the raw sample before HCl treatment. The XRD of the sample after 10 h HCl treatment exhibits two detectable broad diffraction peaks around 26° and 43°, indicating that the carbonaceous structure retains after the removal of MgO by HCl; a small peak around 62° should be assigned to small amount of cubic MgO residue which is wrapped in the graphene sheets and difficult to be washed by hydrochloric acid. According to this pattern, the broader (002) peak may be due to the corrugation and defects structure of the graphene sheets after HCl treatment. The interlayer spacing (d_002_) of the sample are about 0.34 nm, which is slightly larger than the d_002_ spacing of graphite (d_002_ = 0.335 nm)^34^.

The morphology and microstructure of as-prepared samples were imaged by SEM tests. Figure 2b shows the typical SEM image of the raw sample washed by distilled water and absolute ethanol and without acid treatment; it could be clearly observed that the structure is constructed by the core of MgO particles with diameters of 300 ~ 500 nm and interconnected ultrathin graphene sheets wrapped outside. Figure 2c shows the SEM image of the raw sample with 3 h HCl treatment, the MgO particles were partially removed by HCl and the graphene sheets were preserved. Figure 2d displays the sample after 10 h HCl treatment: the MgO template^35^ were totally removed and free-standing nanosheets exhibit a typical wrinkled structure with corrugation and scrolling, which results from thermodynamically stable bending.

The formation process of the graphene nanosheets based on a solution-growth pathway could be deduced from the SEM images (Fig. 2e). Firstly, Mg powder is reacted with H_2_O and HMTA to obtain MgO and the MgO would agglomerate into clusters. Then, the MgO clusters would act as structural constructor, and the curled and overlapped nanosheets are synthesized on the surface of the MgO clusters. When the samples are washed by HCl, the two-dimensional structure of graphene is well maintained and the nanosheets reveal a curled morphology consisting of a thin crumpled paper-like structure.

**Figure 3:**
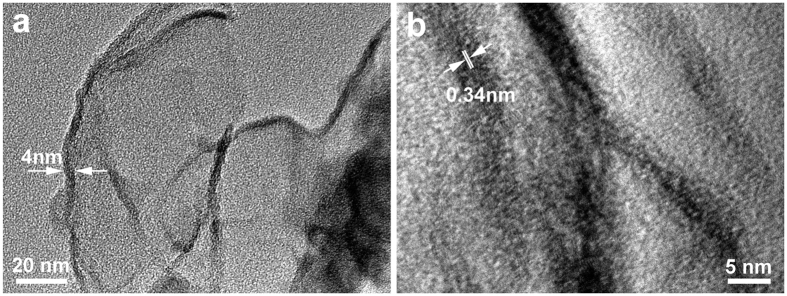
(**a**) TEM images reveal the thin crumpled paper-like structure; (**b**) high-resolution TEM image of a typical graphene sheet.

Figure 3a shows the low magnification TEM images of transparent nanosheets with size of about 100 nm. A typical crumpled surface indicates the features of the high specific surface area and the two-dimensional structure of graphene are well maintained after HCl treatment. The transparency reveals that the sheets consist of graphene with only a few layers. Further high resolution TEM (HRTEM) observation in Fig. 3b reveals that the nanosheet is about 4 nm in thickness which corresponds to approximately 10 stacked monatomic graphene layers. The layer-to-layer distance (d_002_ spacing) is measured to be about 0.34 nm, which is larger than that of graphite (0.335 nm) and agreed with the interlayer spacing calculated by XRD pattern. It is worth noting that the basal planes are discontinuous and distorted, and some parts are wavy and turbostratic, indicating that the layer stacking is disordered^36^, which possibly caused by the uncontrolled hydrothermal reassembling process. The defects in the basal plane could facilitate lithium ion diffusion and storage. Defects on graphene basal plane seem to play an important role in lithium diffusion.

**Figure 4:**
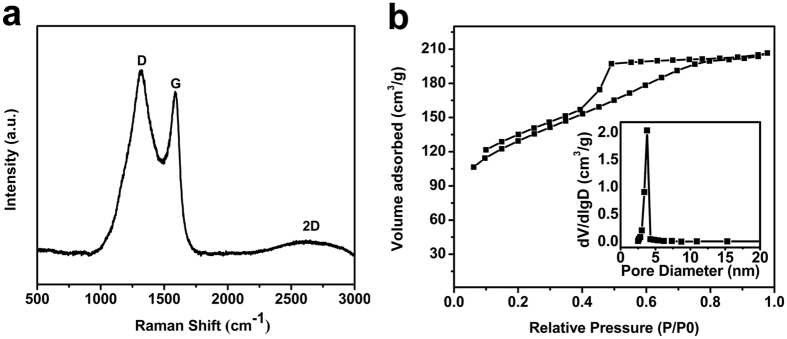
(**a**) Raman spectra of N-doped graphene nanosheets; (**b**) Nitrogen-adsorption isotherms of the N-doped graphene, the inset is BJH desorption pore-size distribution.

Raman spectra have sensitive response to the crystallinity, defects and disorder of microstructure of carbon materials^37^,^38^; so it is carried out as an effective tool to detect the microstructure of the as-prepared sample. As shown in Fig. 4a, the Raman spectrum obtained from N-doped graphene sample shows two first-order Raman peaks centered at 1320 cm^−1^ and 1586 cm^−1^, which could be ascribed to the well-documented D and G band^39^, respectively. The G band originated from the doubly degenerate zone center phonon E_2g_ mode corresponds to ordered *sp2* bonded carbon and provides the formation of graphitic carbon. The D band arises from the breathing modes of six-atom rings of κ-point phonons of A_1g_ symmetry with defects for activation^40^; so the high intensity of the D-band indicates the presence of structural defects in the graphene layer; the defects include boundaries, bonding disorders, vacancies and heteroatoms in graphene lattice generated by nitrogen doping. The second-order 2D band (the broad and weak peak around 2650 cm^−1^) is the D-band overtone^40^ and is a characteristic feature of few-layered graphene^41^,^42^.

The D, G and 2D bands of graphene^43^ provide valuable information of the structural defects; the large D/G ratio and the broadened G and 2D band indicate various bonding structures and defects with small *sp2* domains. The ratio of D to G band integrated intensities (I_D_/I_G_) is usually used to estimate the average crystallite size along the a-axis (L_a_) of *sp2* regions in the graphene layer. The L_a_ could be calculated by the empirical Tuinstra-Koenig equation: L_a_ = (2.4 × 10^−10^) λ^4^(I_D_/I_G_)^−1^ (λ = 514.5 nm,)^37^,^44^. The I_D_/I_G_ is 1.13, so the crystallite size of ordered *sp2* regions surrounded by areas of nitrogened carbon atoms or defects is about 14.9 nm. As a consequence, the Raman spectrum reveals that the N-doped graphene are composed of few-layered structure with sufficient defects which is crucial to improve its lithium diffusion and storage properties.

To further investigate the structure and characterize the porosity of the N-doped graphene, nitrogen adsorption–desorption isotherms were carried out (Fig. 4b). Brunauer-Emmett-Teller (BET) analysis showed that the specific surface area of the N-doped graphene is up to 466 m^2^g^−1^, as well as the high volume of mesopores (0.150 cm^3^/g calculated by the Barrett–Joyner–Halenda (BJH) method). The N-doped graphene exhibits a characteristic type IV isotherm with a pronounced hysteresis at P/P^0^ = 0.4–0.8 between the adsorption and desorption branches, suggesting the existence of a large number of mesopores with uniform pore size distribution^35^. In addition, this type IV isotherm with type H2 hysteresis loop associates with capillary condensation taking place in connecting cage-like mesopores^45^. The hysteresis loop is mainly caused by different mechanisms between capillary condensation and evaporation processes occurring in pores with narrow entrances and large pore interiors with porous network. The capillary evaporation (at P/P^0^ = 0.45) is significantly delayed comparing with the capillary condensation. This delay process^46^ arises from the lack of direct access of the N_2_ condensed in the pore interiors to the exterior when the N_2_ is condensed in the narrower connecting pores^47^. So the volume of the pores^48^ are large enough to show capillary condensation at pressure significantly higher than P/P^0^ = 0.4. In this case, the ink bottle-like pore networks account for the occurrence of broad hysteresis loops. Moreover, the adsorption and desorption branches do not close below P/P^0^ = 0.3, which indicates that chemical adsorption might occur in the mesopores; this chemical adsorption is irreversible so the adsorpted N_2_ could not be desorpted at lower pressure. The pore size distribution (shown in Fig. 4b inset) calculated by BJH method based on the desorption branch displays a unimodal peak centered at 3.8 nm; those pores might be mainly caused by the removal of the template. The existence of the mesopores and interconnections are important for the fast transport of lithium ions and fast access of the electrolyte^49^ because they could provide a more favorable path for penetration and transportation of ions and electrolyte into graphene structure.

**Figure 5:**
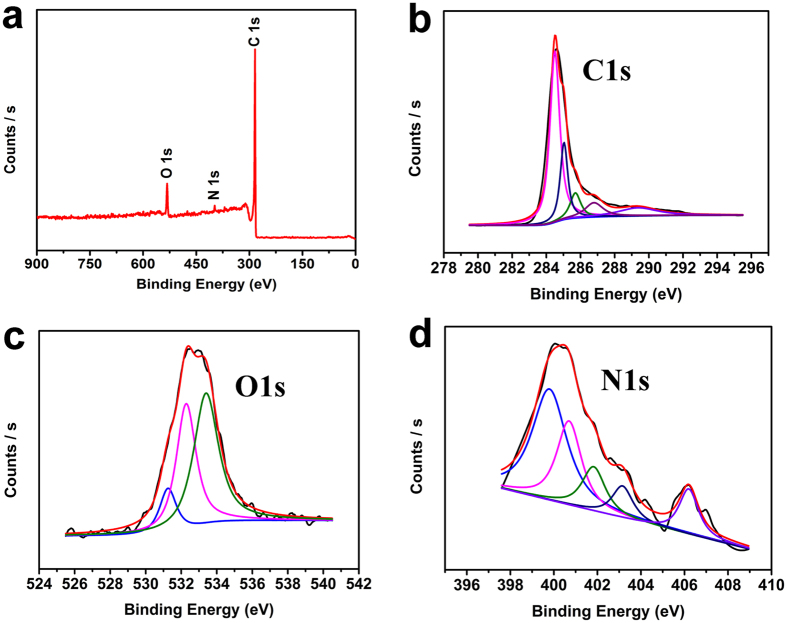
(**a**) XPS survey spectra of the N-doped graphene; (**b**) High resolution C1s XPS spectra; (**c**) High resolution O1s XPS spectra; (**d**) High resolution N1s XPS spectra.

The chemical states of the elements are evaluated by X-ray photoemission spectroscopy (XPS) (Fig. 5). As shown in Fig. 5a, the survey scan shows the existence of graphitic C 1 s peak at 284.6 eV, a weak O 1 s peak at 530 eV, and a pronounced N 1 s peak at 400 eV. XPS of C 1 s ranging from 280–290 eV (Fig. 5b) exhibits six peaks by curve fitting of the C 1 s spectrum. The main peak at 284.5 eV could be assigned to the sp2 carbon atoms (C1) constituted graphitic regions, which demonstrates that most of the C atoms are arranged in a honeycomb lattice. The other five weak peaks centered at 285.6, 285.7, 286.7, 287.9 and 290.3 eV correspond to the sp3 C, C-N, C-O, C=O, and O=C-O groups, respectively^50^, which originate from the groups in the intermediate small molecules which are retained at the edges and defects of the graphene sheets^51^ as showed in the schematic mechanism in Fig. 1. The O 1 s spectrum could be deconvoluted into three subpeaks at 531.2, 532.3 and 533.5 eV (Fig. 5c), which could be attributed to the presence of different oxygen functionalities such as C=O, C-O, and O=C-O, respectively^52^. The N 1 s peak can be split into five individual peaks (as shown in Fig. 5d) located at 399.2 eV, 400.1 eV, 401.4 eV, 402.9 eV and 405.6 eV associated to pyridinic, pyrrolic, graphitic, oxidized and chemisorbed nitrogen, respectively (as showed in the schematic structure of the N-doped graphene in Fig. 1). The amount of nitrogen doped into the graphene is 1.68 at% calculated by XPS elemental analysis. The low doping level could be ascribed to the high reaction temperature, which could break most of the C-N bonds. As shown in Fig. 1, pyridinic and graphitic N present a marginal influence on the graphene structure because the bond lengths of C-N (1.41 Å) and C-C (1.42 Å) are similar^3^; while the pyrrolic N with sp3 bond disrupts the six-atom rings structure of graphene^53^. Moreover, pyridinic N bonding configuration is the most stable structure in the presence of monovacancy, as well as pyridinic and graphitic N show in the presence of divacancy defects^54^. So the occurrence of nitrogen atoms into the honeycomb-like lattice are accompanied with the structure defects such as vacancies, bonding disorders and noncyclized structures, which creates high disorder of the structures. Moreover, due to the higher electronegativity^3^ and smaller covalent radius of nitrogen, the doping would significantly influence the structure and electronic properties of the graphene; and lithium might be favorable for around the defects and sites in the vicinity of residual N atoms.

**Figure 6:**
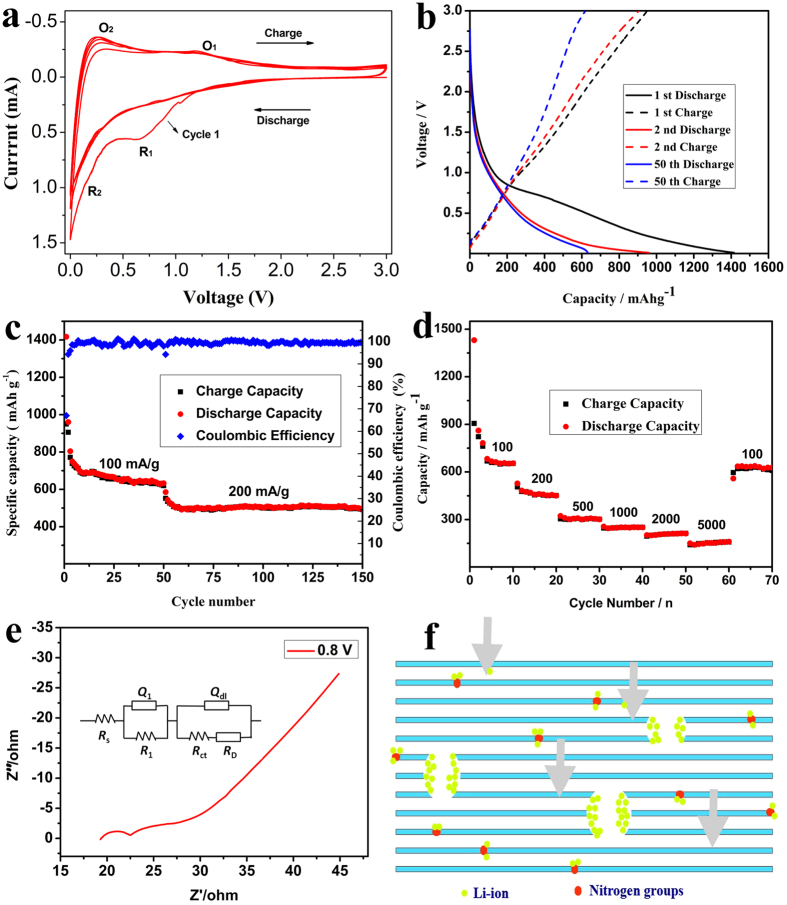
Electrochemical performance of the N-doped graphene electrode: (**a**) Cyclic voltammograms (CV); (**b**) Galvanostatic charge/discharge profile for selected cycles; (**c**) Discharge/charge capacity and coulombic efficiency; (**d**) Rate performance; (**e**) Nyquist plots and equivalent circuit of the first cycle at 0.8 V; (**f**) Proposed scheme describing the Li diffusion mechanism through N-doped graphene, broad down arrows designate Li ion diffusion through defect sites of graphene plane.

Figure 6 shows the electrochemical properties of the N-doped graphene electrode. Cyclic voltammetry (CV) of the first 5 cycles was processed to survey the electrochemical cycling behaviors of the N-doped graphene. The electrode presents two cathodic peaks R_1_ (0.7 V) and R_2_ (below 0.2 V) and two anodic peaks O_1_ (1.2 V) and O_2_ (0.2 V) during the first cycle. The R_1_ peak in the 1st cycle and disappeared in the following cycles could be assigned to the decomposition of the electrolyte and formation of solid electrolyte interphase (SEI) film on the surfaces of graphene in the first cycle^55^. The R_2_ peak and the counterpart anodic O_2_ peak could be attributed to Li ions insertion into the graphene layers^56^. The O_1_ peak indicated that the breaking of the bonds of the Li atom with the defects or other active sites during the charge processes takes place at high voltage^57^. This may be caused by the relatively strong bonds of lithium interactions with the residual nitrogen-containing functional groups within graphene nanosheets. Moreover, the intensity and position of the peaks after the first cycle steadily maintains which indicates that the discharge/charge process is stable. After the second cycle, the current peaks became stable, which indicates that the insertion/extraction of Li^+^ produces good reversibility.

Galvanostatic charge/discharge of the sample was measured at a current rate of 100 mA/g in the potential range from 0.01 V to 3 V with Li foil as a counter electrode at room temperature (25 °C). Figure 6b shows the typical charge/discharge profile for selected cycles. The intercalation of Li^+^ of the first cycle begins at around 2.0 V vs. Li/Li^+^; the curve exhibits a slope plateau at 0.8 V which arises from the combination of Li ions adsorption on the structural defects in basal planes of the graphene layers and the formation of solid electrolyte interphase (SEI) film^58^,^59^ by the electrolyte decomposition reactions on the surfaces of graphene. No distinct plateau below 0.3 V (the feature of lithium staging^56^,^60^ in carbon layers) could be observed, suggesting the disordered structure providing electronically and geometrically nonequivalent sites for lithium storage. The initial discharge and charge specific capacities are up to about 1420 mAh/g (equivalent to ~0.63 mol Li per mol C) and 950 mAh/g (~0.42 mol Li per mol C), respectively. The extra capacity over the theoretical specific capacity originates from the large amount of surface defects and high surface area for lithium storage. After the first cycle, the discharge curve changes into a steep slope during the discharge/charge process, suggesting that the formation of the SEI film only occurs in the first cycle. The discharge capacity of the second cycle decreases to 960 mAh/g. The irreversible capacity loss of 460 mAh/g could be caused by both electrolyte decomposition on graphene surface and strong Li ions adsorption^61^ on the special positions like vacancies or vicinity of residual N groups.

Figure 6c presents the discharge/charge cycling performance of the N-doped graphene electrode at 100 mA/g. After the fifth cycle, the specific capacity is stable and maintains at above 600 mAh/g (0.27 mol Li per mol C) after 50 cycles, indicating high capacity retention of the electrode. When the current density raised up to 200 mA/g, the stationary capacity still keeps above 500 mAh/g which is significantly higher than the theoretical capacity of graphite after 150 cycles. The columbic efficiency of the first cycle is about 67% corresponding to the large irreversible capacity in the initial discharge/charge process. Then the columbic efficiency increased up to above 98% in the following cycles. Besides the excellent cyclability, the rate capacity of N-doped graphene is also measured at different current densities (Fig. 6d). The current intensity increases stepwise from 100 to 200, 500, 1000, 2000 and 5000 mA/g, and the corresponding stable discharge capacities are 655, 453, 306, 259, 201 and 150 mAh/g, respectively. When the current goes back to 100 mA/g, the capacity could return to ~650 mAh/g, which almost recovers the initial capacity, indicating that the special structure of the graphene keeps stable at the high current density.

The above analysis well demonstrated that N-doped graphene is an excellent electrode material of Li-ion battery. To get further insight into the electrochemical process, electrochemical impedance spectroscopy (EIS) were carried out by three electrode system and the obtained Nyquist plots are presented in Fig. 6e. The impedance data could be simulated by the appropriate electric equivalent circuit in the inset of Fig. 6e. The circuit includes ohmic resistance (R_s_), resistance (R_1_) of the surface film formed on the electrodes and the contact problems^62^, double layer capacitance (Q_dl_), charge-transfer resistance (R_ct_)^38^, and the Warburg impedance (R_D_) related to the diffusion capacitance of lithium ions into the graphene electrodes^63^. It could be calculated that the electrode possesses low charge transfer resistances (Rs = 19.64 Ω, R_1_ = Ω and R_ct_ = 2.738 Ω). In this case, the electrical conductive capability are improved, resulting in the high capacity and stable cycling performance.

According to the above results, the significantly improved electrochemical performance of N-doped graphene get benefit from the unique structure by nitrogen doping^39^, because it introduces a large number of topological defects on the graphene layers, which leads to the formation of disordered carbon structure that further improves the lithium storage capacities. As shown in Fig. 6f, the existence of multiple lithium storage positions such as N-groups, grain boundaries, vacancies and mesopores increase the reversible capacity of the N-doped graphene. First, doping plays an important role in promoting the electrochemical performance, because N-doping effect may offer favorable active sites^61^,^64^ around the vicinity of residual N-groups for lithium storage even though such a process may occur at high equilibrium voltages and high overvoltages. Second, the numerous mesopores (3.8 nm) produced during the fabrication process offers optimized conditions for electrolyte penetration and facile transport channels for lithium ions migration especially at high rate^60^. In addition, the open pores and vancancies could serve as active intercalation sites for lithium ions contributing to the high capacity, though a large irreversible capacity loss is concomitant as well. Moreover, the conductive nanosheets with large surface area greatly reduce the solid-state transport lengths for lithium ion diffusion and guarantee a continuous electronic pathway. The high specific surface area also provides a high electrode/electrolyte contact interface to facilitate fast charge transfer reaction and minimize polarization effects which is benefit to the stability of the electrode. The above features and their multiple synergistic effects make N-doped graphene a favorable candidate electrode material with superior rate capability, high capacity and cycle performance.

## Discussion

In summary, we have successfully prepared a new kind of N-doped graphene through a facile one-step hydrothermal approach. The as-synthesized graphene with incorporation of nitrogen groups possesses unique structure including high surface area, mesopores and vacancies, which is particularly important for the reversibly storage of lithium ions. The structural features and their synergistic effects could not only promote ions and electrons transportation with nanometer-scale diffusion distances but also benefit to the penetration of electrolyte. The N-doped graphene exhibits outstanding electrochemical properties such as high reversible capacity, superior rate capability as well as long-term cycling stability, which endow it great potential to be an efficient electrode material candidate, and the EIS test further indicates that the N-doped graphene is a good lithium ion battery. The experimental results provide a novel hydrothermal route to fabricate N-doped graphene material with potential application for advanced energy storage, as well as useful information to design new graphene materials for future applications.

## Methods

### Synthesis of Nitrogen-doped Multilayer Graphene

All reagents (purchased from Shanghai Chemical Reagents Company) were analytical purity and used without further purification.

Details of a typical synthesis process are as follows: Hexamethylenetetramine (HMTA, 3.0 g), magnesium powder (200 mesh, 4.0 g) and ultrapure water (3 mL) were added into a 20 ml stainless steel autoclave. The autoclave was sealed, put in an electric furnace, warmed up at a rate of 10 °C/min and maintained at 500 °C for 20 h, and was then cooled to room temperature naturally. The precipitate was filtered off, washed with distilled water, absolute ethanol, and hydrochloric acid solution (5 mol/L) for several times, and then dried in vacuum at 50 °C for 3 h.

### Sample Characterization

The X-ray powder diffraction (XRD) measurements were carried out on a Bruker AXS D8 Advance X-ray diffractometer equipped with Cu Ka radiation (λ = 1.54182 Å) at a scanning rate of 10° min^−1^. The morphology of the graphene were observed using a JEOL JSM-6700F field emission scanning electron microscope (SEM) operated at 15 kV and Hitachi H7650 transmission electron microscope (TEM) operated at 100 kv. The high resolution images were recorded using a high resolution transmission electron microscope (HRTEM, JEOL-2010) operated at 200 kV. The surface areas of the sample were measured by TriStar II 3020 (Micromeritics Instrument Corporation, U.S.A.) and calculated by N_2_ adsorption-desorption isotherms. The XPS tests were performed on a Thermo Scientific ESCALAB 250 X-ray photoelectronic spectrometer with a non-monochromated Mg Kα X-ray radiation as the excitation source. The Raman spectroscopy data were taken on a LABRAM-HR laser MicroRaman spectrometer with excitation wavelength of 514.5 nm (2.41 eV).

### Electrochemical Measurements

The electrochemical performances were measured with CR2032 coin-type cells. The N-doped graphene acted as an anode electrode, and Li foil was used as a counter electrode. To prepare the working electrodes, the active materials (70 wt %) were mixed with Super P carbon black (20 wt %) and polyvinylidene fluoride (PVDF) (10 wt %). These materials were dissolved in N-methyl pyrrolidone (NMP) and ball milled for 5 hours at speed of 400 rpm to obtain uniform slurry. Then the slurry was coated on a Cu foil current collector and subsequently dried under vacuum at 80 °C for 12 h. The Celgard 2300 microporous membrane was used as polypropylene separator, and the electrolyte was a 1 mol/L solution of LiPF_6_ in ethylene carbonate/dimethyl carbonate (EC/DMC) with a volume ratio of 1:1. The galvanostatic charge/discharge tests were tested in the voltage range of 0.01–3.0 V using a computer-controlled multichannel battery test unit (LAND-CT2001A battery cycler) at room temperature. The typical cyclic voltammetry (CV) measurements were performed in the potential window of 0.01–3.0 V at a scanning rate of 0.1 mV/s on LK-2005A electrochemical workstation.

## Additional Information

**How to cite this article**: Xing, Z. *et al.* One-pot hydrothermal synthesis of Nitrogen-doped graphene as high-performance anode materials for lithium ion batteries. *Sci. Rep.* **6**, 26146; doi: 10.1038/srep26146 (2016).
